# Adherence to the Mediterranean Diet in a Portuguese Immigrant Community in the Central Valley of California

**DOI:** 10.3390/nu13061989

**Published:** 2021-06-09

**Authors:** Roberto M. Couto, Andrew D. Frugé, Michael W. Greene

**Affiliations:** Department of Nutrition, Dietetics, and Hospitality Management, Auburn University, Auburn, AL 36849, USA; rmc0060@auburn.edu (R.M.C.); adf0003@auburn.edu (A.D.F.)

**Keywords:** Mediterranean diet, adherence, Portuguese immigrants, California

## Abstract

The Mediterranean Diet (MedDiet) is a healthy eating pattern associated with a better quality of life among older adults and reduced risk of non-communicable diseases. Little is known about the MedDiet in immigrant communities from countries in which the MedDiet is a settled cultural heritage. Thus, we examined MedDiet adherence and perceived knowledge, benefits, and barriers to the MedDiet in a Portuguese immigrant community in Turlock, California. A cross-sectional study was conducted with 208 participants in Turlock and Livermore, California, which was used as a reference population. Univariate, multivariable, and logistic regression models were used for data analysis. Compared to the Livermore group, the Turlock group was younger and less educated, but had a higher average MedDiet score and active adherence to a MedDiet (*p* < 0.001 for both). In the Turlock group, convenience, sensory appeal, and health were observed to be significant barriers to the MedDiet (*p* < 0.05), while health, weight loss, natural content, familiarity, price, sensory appeal, and mood were significant benefit factors (*p* < 0.05). In conclusion, participants in Turlock had greater MedDiet adherence despite lower education attainment. Furthermore, the perceived benefits of the MedDiet were key factors in MedDiet perception and adherence in a Portuguese immigrant community.

## 1. Introduction

Nutrition is thought to be a core element in ameliorating health conditions in a globally aging society [1]. The Mediterranean Diet (MedDiet) has been widely reported as a model of a healthy eating pattern for a better quality of life among older adults [2,3] and for reducing the risk of the most prevalent non-communicable diseases associated with aging [4], such as cardiovascular disease [5,6,7], cancer [8], metabolic syndrome [6], obesity [5,6], and dementia [3]. The MedDiet is not universally defined because it is a dietary pattern from a relatively heterogeneous group of countries bordering the Mediterranean Sea and also within the countries themselves [6]. However, the traditional MedDiet is a plant-based diet that is characterized by high consumption of fruits, non-refined cereals, vegetables, olive oil, and nuts; a moderate amount of chicken and fish, and lower consumption of dairy, red meat, and sugars; use of aromatic herbs; water as the primary beverage; and wine in moderation [9].

Portugal is the most western country in southern Europe and is geographically not in the Mediterranean basin. Still, the Mediterranean diet is a settled cultural heritage of the Portuguese population and cuisine [10], with ancestral food-related influence from their Mediterranean neighbors and specificities from surrounding migrants [11,12]. MedDiet adherence in Portugal was similar to that of Spain for the period of 2004–2011 when assessed from Food and Agriculture Organization Food Balance Sheets [13]. Like Italy, Greece, and Spain, MedDiet adherence in Portugal was greatly reduced from 1961 to 1965 compared to 2004–2011 [13]. As observed in neighboring countries, regional differences in adherence to the Mediterranean food pattern exist in Portugal. The regions of Algarve and Madeira have the highest MedDiet adherence, while Lisbon and Tejo valley and the Azores have the lowest [14].

Portuguese immigration to the United States occurred in two major waves: the first from 1890 to 1930 and the second from 1950 to 1990 [15]. Approximately 1.37 million Portuguese Americans now live in the United States [16]. The majority of Portuguese immigrants were from the Azores [17] and settled primarily in Massachusetts and California, mainly in the Central Valley [15,17,18]. The Azores region of Portugal has one of the least educated populations in Portugal and is one of the poorest regions in Portugal [19,20]. Acculturation of Portuguese immigrants has been shown to be similar to that of Greek immigrants, yet Portuguese immigrants have historically had lower educational attainment and a slightly higher poverty rate [15]. Assessing this population’s adherence to the MedDiet and the factors influencing these behaviors can provide meaningful insight from a public health perspective.

The application of behavior change models to diet can assist in determining appropriate measures for nutrition assessment, intervention, and outcome evaluation as well as the adoption of healthy behaviors and diets [21]. The transtheoretical model of behavior change focuses on behavior change being a dynamic process occurring in the following stages and processes of change (unaware, unengaged, deciding, decided no, decided yes, action, and maintenance) [22]. An individual’s stage of change is influenced by beliefs, experiences, prior knowledge, and perceived benefits and barriers towards their behavioral change [23,24]. Perceived benefits and barriers towards adopting a dietary approach can be strong predictors of an individual’s food choice and the individual’s willingness to alter or adjust their current lifestyle [25].

Even though MedDiet adherence has previously been assessed in populations living throughout the world [2], including elderly Portuguese [26] and the United States [27,28,29,30,31], few studies have examined MedDiet adherence in communities with immigrants from countries in which the MedDiet is a settled cultural heritage. The current study aimed to assess MedDiet adherence and its associated factors in a Portuguese immigrant community in the Central Valley of California, USA.

## 2. Materials and Methods

### 2.1. Study Setting

The present study was conducted at two locations in Central California. Livermore, with a population of 90,189 [17] located in Alameda County, is the easternmost city in the San Francisco Bay Area and is considered a gateway community to the Central Valley. Livermore is a science and technology center that contains the Lawrence Livermore National Laboratory and Sandia National Laboratories and has a median household income of USD 116,942 [32]. In Livermore, 77.2% of the population is white, 1.8% African American, 11.1% Asian, and 20.2% Hispanic or Latino, 0.2% American Indian and Alaska Native [17]; 51.1% of the population is female [17]; 12.9% of the population is 65 years old and over, while 23.5% are under the age of 18; and 41.8% of the population holds a bachelor’s degree or higher [17]. According to the United States Census American Community Survey, the top three reported ancestries reported in Livermore city are German, Irish, and English [33].

With a population of 73,631, Turlock is the second largest town in Stanislaus County, located in the rural San Joaquin Valley region of the Central Valley [32]. Turlock has a median household income of USD 56,639 [32]. Turlock contains one public university, California State University Stanislaus [18]. In Turlock, 76.8% of the population is white, 2.4% African American, 5.6% Asian, 37.1% Hispanic or Latino, 0.7% American Indian and Alaska Native [18]; 51.9% of the population is female; 13.0% of the population is 65 years old and over, while 26.8% are under the age of 18; and 24.5% of the population holds a bachelor’s degree or higher and 81.1% hold a high school education or higher [18]. According to the United States Census American Community Survey, the top three reported ancestries reported in Turlock city are German, Portuguese, and English [33].

### 2.2. Survey Instrument

A previously validated survey instrument was used to assess MedDiet adherence, participants’ stage of change, barriers, and benefits towards adopting the MedDiet and demographic variables [29]. Participants’ MedDiet adherence was assessed using a validated [34] 14-question Mediterranean Diet Adherence Screener (MEDAS) that has been used to evaluate MedDiet adherence in Europe, including Portugal, and throughout the world [27,35,36,37]. Additionally, three questions were asked to assess participants’ readiness to adopt a MedDiet using the Precaution Adoption Process Model (stages of change). Perceived barriers (18 questions; knowledge, convenience, sensory appeal, and health) and benefits (26 questions; knowledge, weight loss, ethical concerns, natural content, familiarity, price, sensory appeal, and mood) to the MedDiet were measured using a five-point Likert scale. Seven demographic and anthropometric questions determining the age, sex, ethnicity, height, weight, level of education, and previous nutrition education or knowledge were assessed. Body Mass Index (BMI) was calculated by dividing weight in kilograms (kg) by height in meter (m) squared. All survey questions were self-reported. The survey instrument was translated from English to Portuguese by a Portuguese language teacher and was back-translated from Portuguese to English by three native Portuguese speakers.

### 2.3. Survey Distribution

This study was approved by the Auburn University Institutional Review Board prior to distributing the surveys, which included language for inferred consent. Convenience sampling was used to obtain completed surveys from shoppers of Save Mart Supermarket stores in both Livermore and Turlock, California, from 14 October 2019 to 1 January 2020. Both Livermore and Turlock Save Mart Supermarket stores were open seven days a week, but only allowed sampling and data collection on Mondays and Saturdays. All Save Mart Supermarket Corporation rules and regulations for outside vendors were followed. Participants were not compensated for completing the survey instrument. All adults at least 45 years old in Livermore and Turlock were eligible for the study. Participants were provided the option of completing the survey in Portuguese. Approximately 37% of the participants residing in Turlock completed the survey in Portuguese. Two hundred and eleven participants completed surveys. Three surveys were excluded because participants did not answer all questions or did not meet the age requirement to participate in the study. The remaining 208 responses were a priori divided into two groups based on the geographical location of survey collection in California: Turlock (*n* = 125) and Livermore (*n* = 83).

### 2.4. Statistical Analyses

All data analyses were conducted with RStudio and the Rx64 3.6.0 software environment (RStudio, PBC, Boston, MA, USA). A crude (unadjusted) and multivariable backward stepwise linear regression analysis was used to assess the differences in total MedDiet adherence scores between the groups. Regression coefficient *p* values and main effect *p* values calculated using a type III Sum of Squares test were reported. A multivariate linear model was used to determine barriers and benefit question scores in the groups: the crude model was unadjusted and the adjusted model included all demographic variables. A backward stepwise elimination logistic regression was performed to identify the predictors of the stage of change with the demographic variables. Inclusion and retention criteria in the logistic regression model were set at *p*-value cutoff points of 0.25 and 0.10, respectively. A significance level of 0.05 was established.

## 3. Results

### 3.1. Demographic Assessment

We examined whether there were demographic differences between participants in the Turlock and Livermore groups. As shown in Table 1, significant differences (*p* < 0.05) in age, ethnicity, and education were observed between the participants in Turlock compared to Livermore. The Turlock group had a lower percentage of older adults (>65 years old), a greater percentage of participants in the ‘other ethnic group’ category, and a lower percentage of participants with associate’s, bachelor’s, and master’s/profession degrees. There were no statistical differences between groups for sex, BMI, and health-related qualifications.

### 3.2. Mediterranean Diet Adherence

The total (MEDAS) score was analyzed using a crude and multivariable backward stepwise linear regression model adjusting for the demographic variables of sex and age. We observed an increase in the MEDAS scores in the Turlock group in comparison to the Livermore group in both the crude and adjusted models (Table 2). For each point increase in the MEDAS score in the Livermore group, an increase of 0.85 +/− 0.26 points (*p* = 0.001) was observed in the Turlock group in the crude model and 0.81 +/− 0.30 points (*p* = 0.002) in the adjusted model. When evaluating both demographic variables and MEDAS score, the MEDAS score was 0.55 +/− 0.25 points lower in males than females and 5.48 +/− 1.79 points higher in respondents older than 75. The demographic variables of education, BMI, or nutrition qualifications were not significant and did not improve the parsimoniousness of the lineal model.

### 3.3. Barriers to Consuming a MedDiet in Turlock

Cronbach’s alpha was calculated to assess the internal consistency of 18 questions sorted into four factors: knowledge, convenience, sensory appeal, and health. As shown in Table 3, The Knowledge barrier had a Cronbach’s alpha = 0.43, which is below the 0.60 to 0.70 that is considered acceptable or adequate for assessing the internal consistency [38]. We did not remove questions to improve the reliability of the Knowledge barrier. The Convenience (Cronbach’s alpha = 0.76), Sensory Appeal (Cronbach’s alpha = 0.75), and Health barriers (Cronbach’s alpha = 0.87) were internally valid. We used both an unadjusted (crude) and an adjusted linear regression model for sex, age, ethnicity, education, and BMI to assess knowledge, convenience, sensory appeal, and health barriers in the Turlock group compared to the Livermore group. Convenience (β = 1.12, SE = 0.50, *p* = 0.027), Sensory Appeal (β = 0.69, SE = 0.33, *p* = 0.041), and Health (β = 0.39, SE = 0.13, *p* = 0.003) were observed to be significant barriers to the MedDiet in the Turlock group (Table 3).

### 3.4. Benefits to Consuming a MedDiet in Turlock

Health, Weight Loss, Ethical Concerns, Natural Content, Familiarity, Price, Sensory Appeal, and Mood factors were used to assess the perceived benefits of adopting a MedDiet among survey participants. All eight barrier factors were internally valid (Health = 0.93; Weight Loss = 0.63; Ethical Concerns = 0.89; Natural Content 0.61; Familiarity = 0.80; Price = 0.91; Sensory Appeal = 0.79; Mood = 0.93). Similar to the assessment of barriers, we used both unadjusted and adjusted linear regression models for sex, age, ethnicity, education, and BMI to assess the benefits from adopting a MedDiet in the Turlock group using the Livermore group as a reference (Table 4). The Turlock group perceived the MedDiet to have more health benefits in the adjusted model (Health: β = 3.72, SE = 0.98, *p* = <0.001), and this association remained consistent in the crude model. Additionally, Familiarity (Familiarity: β = 0.99, SE 0.26, *p* = <0.001) was perceived to be a benefit in the Turlock group in both regression models. All benefit factors, except ethical concerns in the adjusted models, were significant (*p* < 0.05) across the three models in the Turlock group compared to the Livermore group.

### 3.5. Stages of Change and Demographic Influences

The distribution of participants across stages of change between the Turlock and Livermore groups was significantly different (*p* < 0.001) (Table 5). More participants in the Action/Maintenance category were observed in the Turlock group. In contrast, the Livermore group had more participants in the Unaware/Unengaged, Deciding, and Decided No category than the Turlock group (*p* < 0.001) (Table 5).

We performed logistic regression to examine the effect of demographic variables on the likelihood of being in each stage of change towards adopting the MedDiet in the whole cohort (Table 6). Participants were significantly less likely to be in the Unengaged/Unaware stage if they were from the Turlock cohort (OR = 0.23, 95% CI: 0.11–0.45, *p* = <0.001). Additionally, participants who had a master’s or professional degree were shown to be less likely to be in the Unaware/Unengaged stage of change (OR = 0.20, 95% CI: 0.05–0.63, *p* < 0.01). Participants categorized as Black (OR = 9.27, 95% CI: 1.50–180, *p* = <0.05) were significantly more likely to be in the Unaware/Unengaged stage. In regard to the Deciding and Deciding Yes groups, no significant associations were observed. In contrast, participants with a GED education were more likely to be in the Deciding No stage of change (OR = 9.36, 95% CI: 1.13–65.3, *p* <0.05). Lastly, the Turlock cohort was significantly more likely to be in the Action/Maintenance group (OR = 17.7, 95% CI: 6.46–64.6). However, participants who are classified as having a master’s or professional degree (OR = 5.59, 95% CI: 1.71–21.0) were more likely to be in the Action/Maintenance group.

## 4. Discussion

MedDiet adherence and related factors affecting adherence have not previously been studied among Portuguese immigrants in the United States. Therefore, we used a recently developed survey instrument to assess participants’ MedDiet adherence, the participants’ stage of change towards incorporating the MedDiet in their lifestyle, and perceived benefits and barriers to consuming a MedDiet [29]. To ensure the inclusion of Portuguese adult immigrants in the Turlock group, we surveyed adults older than 45 years old at the local Save Mart store in Turlock and provided the participants the opportunity to complete the survey in Portuguese. Indeed, 37% of the Turlock participants preferred to take the survey in Portuguese, while none of the participants identified as Portuguese in Livermore. The population in the present study had a greater percentage of female respondents than reported in the Livermore and Turlock communities [17]. In contrast, the Livermore population in the present study had comparable ethnicity and education demographics compared to those in the Livermore community [17]. In the Turlock population in the present study, comparable ethnicity but not education (less participants in the study with holding a bachelor’s degree) to that in the Turlock community [17] was observed.

We found that the Turlock group had higher adherence to the MedDiet as assessed by the MEDAS score compared to the Livermore group. Similarly, a higher percentage of participants in the Turlock group were in the Action/Maintenance stage of change compared to the Livermore group. This result was surprising given that the majority of respondents in the Turlock group had a high school education or lower and that educational level has been shown to be negatively associated with MedDiet adherence in studies performed in the United States [29] and countries bordering the Mediterranean Sea [39,40]. In contrast, our results are consistent with a study in Portugal where Portuguese households from lower social classes had greater adherence to the Mediterranean food pattern compared to participants at higher social levels [14]. Our findings of higher MedDiet adherence scores in Turlock could be due to the “Healthy Immigrant Effect” where immigrants are, on average, healthier than native-born participants in a given population [41]. Whether diet knowledge, diet decision making, or the ability of immigrant families to overcome diet barriers are factors [42] contributing to greater adherence to the MedDiet in the Turlock group requires formal examination. Even though Turlock participants were significantly more likely to be in the Action/Maintenance stage of change, we did observe that approximately one-third of participants in Turlock were in the Unaware/Unengaged stage of change. Whether exposure to dietary acculturation, lack of interaction with family or support groups, length of years spent in the United States, immigration status (first vs. second-generation immigrant), or other social/environmental factors that influence acculturation [42,43] explain our findings with Turlock participants in the Unaware/Unengaged stage of change requires further investigation.

The perceived benefit of Familiarity and perceived barriers of Convenience and Sensory Appeal scores in the Turlock group were significantly higher than those in the Livermore group. The difference in Familiarity may be due to the Turlock participants’ knowledge of the Portuguese cultural cuisine (high fish, fruit, and cheese consumption, moderate wine consumption, and low processed food consumption [44]) which are aligned with most of the dietary components of the MedDiet [45,46], while the differences in Convenience and Sensory Appeal scores in the Turlock group may be due to an inability to obtain culturally familiar and fresh food in an impoverished rural region of California. Our findings are consistent with a prior study in Israel examining Ethiopian immigrants’ perceived benefits of diet and sensory responses while attempting to maintain current dietary patterns, which found that immigrants’ choices are both guided through convenience and familiarity [47]. Sensory appeal and convenience have also been found to be significant factors related to the unwillingness of patients with nonalcoholic fatty liver disease in a Northern European community to adjust their diet patterns to a Mediterranean diet [48].

A surprising result in perceived benefits was that weight loss was seen as more of a benefit in the Turlock group rather than the Livermore group. This result is not consistent with prior findings that weight loss as a perceived benefit was observed in participants with low MedDiet adherence [29]. Whether our current findings on perceived weight loss are specific for immigrants from countries where the Mediterranean diet is a settled cultural heritage will require future studies. Our findings that participants with a Master’s or Professional Degree were over five times more likely to be in the Action/Maintenance stage and had a lower OR of being in the Unaware/Unengaged stage and that participants were nine times more likely to be in the Decided No category if the participant had a GED are consistent with previous studies showing a strong correlation between education level and MedDiet adherence [27,29,49,50,51].

While we took a systematic approach using validated questionnaires to conduct this study, several limitations are acknowledged. We recruited a convenience sample from one Portuguese immigrant population and one reference city; thus, in addition to our relatively small sample size, additional studies with larger Portuguese immigrant populations in other geographic locations are needed to confirm our findings. Similarly, our sample was restricted to adults aged 50 years and older; therefore, the results cannot be applied to the general adult population. Another possible limitation is that the self-reported data in our study, which include weight, height, and dietary assessment, may not reflect actual values. Lastly, we did not formally test the association between immigrant status and high MedDiet adherence in immigrant groups in the United States.

## 5. Conclusions

The present study identifies a significant increase in MedDiet scores between participants in Turlock and participants in Livermore, which was consistent with a greater proportion of Turlock participants in the Action/Maintenance stage of change. Although high educational levels are strongly associated with higher adherence to the MedDiet, the Turlock group had lower education attainment than the Livermore group, yet had greater MedDiet adherence. Importantly, the benefits of health, weight loss, natural content, familiarity, price, sensory appeal, and mood are key factors in the perception of the MedDiet in the Turlock group. In contrast, convenience, sensory appeal, and health were observed to be significant barriers to the MedDiet in the Turlock group.

## Figures and Tables

**Table 1:** Demographics of participants in Turlock and Livermore.

	Turlock ^†^ (*n* = 125)		Livermore ^†^ (*n* = 83)		
	*n*	%	*n*	%	*p*-Value
Sex *					0.724
Male	51	41	31	38	
Female	74	59	52	62	
Age *					**0.004**
45–54	17	13	5	6	
55–64	54	43	21	26	
65–74	39	31	34	41	
>75	16	12	22	27	
Ethnicity *					**0.010**
White	104	83	60	74	
Black	5	4	4	5	
Chinese	0	0	9	9	
Asian-other	0	0	1	1	
Other ethnic group	17	13	9	11	
Education *					**<0.001**
High School or lower	61	61.8	27	26.8	
GED	10	11.4	4	3.7	
Technical or trade certificate	6	5.7	9	8.5	
Associate’s degree	6	5.7	24	24.4	
Bachelor’s degree	8	7.3	27	26.8	
Master’s or professional degree	10	8.1	9	9.8	
BMI *					0.166
Underweight	1	0	2	2.4	
Normal weight	39	31.7	27	40.2	
Overweight	57	43.1	26	24.4	
Obese	29	25.2	27	32.9	
Qualification *					0.321
Health or nutrition related qualifications	3	1.6	5	6.1	
No health or nutrition related qualifications	123	98.4	77	93.9	

* Significance across score categories by Pearson’s chi-squared test; bold font indicates *p* < 0.05; † Turlock, California and Livermore, California. Demographic categories are indicated using grey background.

**Table 2:** Multivariable linear regression analysis assessing Mediterranean diet adherence between groups adjusted for demographic categories, stages of change, barriers, and benefits. Linear regression analysis using a crude and multivariable backward stepwise model to assess Mediterranean diet adherence in the Turlock group.

					Main Effects
		β	SE	*p*-Value *	*p*-Value ^‡^
Crude Model					
Group	Livermore	Ref ^†^			
	Turlock	0.85	0.26	**0.001**	
Backward Stepwise Model					
Group					**0.002**
	Livermore	Ref ^†^			
	Turlock	0.81	0.30	**0.002**	
Sex					**0.030**
	Female	Ref ^†^			
	Male	−0.55	0.25	**0.030**	
Age					**0.043**
	45–54	Ref ^†^			
	55–64	0.14	0.34	0.737	
	65–74	−0.05	0.43	0.901	
	>75	5.48	1.79	**0.002**	

^†^ Ref, reference group; * regression coefficient *p* value; ^‡^ Main effects were assessed by ANOVA using a type III Sum of Squares method; *p* values < 0.05 are indicated in bold font. Variable categories are indicated using grey background.

**Table 3:** Crude and adjusted linear analysis of perceived MD barriers.

	Crude ^†^			Adjusted ^††^		
Barrier	β	SE	*p*-Value *	β	SE	*p*-Value *
Knowledge (*n* = 4) ^‡^ (Cronbach’s Alpha = 0.43)						
Livermore ^∇^	Ref			Ref		
Turlock	0.49	0.37	0.187	0.75	0.43	0.086
Convenience (*n* = 4) (Cronbach’s Alpha = 0.76)						
Livermore	Ref			Ref		
Turlock	1.38	0.44	**0.001**	1.12	0.50	0.027
Sensory Appeal (*n* = 3) (Cronbach’s Alpha = 0.75)						
Livermore	Ref			Ref		
Turlock	0.87	0.30	0.004	0.69	0.33	**0.041**
Health (*n* = 4) (Cronbach’s Alpha = 0.87)						
Livermore	Ref			Ref		
Turlock	0.59	0.40	0.147	0.39	0.13	0.003

^‡^ Number of questions in each factor; * *p* values < 0.05 from type III Sum of Squares method are indicated in bold font; ^†^ Crude linear model; ^††^ Adjusted linear model for sex, age, ethnicity, education, and BMI; ^∇^ Livermore was used as the reference (Ref) group in the linear model.

**Table 4:** Crude and adjusted linear analysis of perceived MD benefits.

	Crude ^†^				Adjusted ^††^	
Benefits	β	SE	*p*-Value *	β	SE	*p*-Value *
Health (*n* = 10) ^‡^ (Cronbach’s Alpha = 0.93)						
Livermore^∇^	Ref			Ref		
Turlock	3.89	0.84	**<0.001**	3.72	0.98	**<0.001**
Weight Loss (*n* = 2) (Cronbach’s Alpha = 0.63)						
Livermore	Ref			Ref		
Turlock	0.78	0.19	**<0.001**	0.74	0.22	0.001
Ethical (*n* = 2) (Cronbach’s Alpha = 0.89)						
Livermore	Ref			Ref		
Turlock	0.30	0.13	0.018	0.20	0.34	0.560
Natural Content (*n* = 2) (Cronbach’s Alpha = 0.61)						
Livermore	Ref			Ref		
Turlock	0.57	0.18	0.001	0.66	0.20	**0.001**
Familiarity (*n* = 2) (Cronbach’s Alpha= 0.80)						
Livermore	Ref			Ref		
Turlock	0.98	0.21	**<0.001**	0.99	0.26	**<0.001**
Price (*n* = 2) (Cronbach’s Alpha = 0.91)						
Livermore	Ref			Ref		
Turlock	0.56	0.22	**0.013**	0.58	0.26	**0.037**
Sensory Appeal (*n* = 2) (Cronbach’s Alpha = 0.79)						
Livermore	Ref			Ref		
Turlock	0.53	0.22	**0.017**	0.56	0.26	**0.041**
Mood (*n* = 3) (Cronbach’s Alpha = 0.93)						
Livermore	Ref			Ref		
Turlock	1.11	0.34	0.002	1.01	0.42	**0.017**

^‡^ Number of questions in each factor; * *p* values < 0.05 from type III Sum of Squares method are indicated in bold font; ^†^ Crude linear model; ^††^ Adjusted linear model for sex, age, ethnicity, education, and BMI; ^∇^ Livermore was used as the reference (Ref) group in the linear model.

**Table 5:** Percent of participants in the Livermore and Turlock groups by stage of change.

Stages of Change	Livermore	Turlock
Unaware/Unengaged	67.1	37.4
Deciding	17.0	4.9
Decided No	6.1	0.8
Decided Yes *	4.9	6.5
Action/Maintenance *	4.9	49.6

* Significance across score categories by Pearson’s chi-squared test (*p* < 0.05).

**Table 6:** Backward stepwise elimination logistic regression of stage of change by demographic factors.

			Stages of Change		
	Unaware/Unengaged	Deciding	Decided Yes	Decided No	Action/Maintenance
	OR (95% CI)	OR (95% CI)	OR (95% CI)	OR (95% CI)	OR (95% CI)
Group					
Turlock	0.23 (0.11–0.45) ***			0.17 (0.20–0.88)	17.7 (6.46–64.6) ***
Sex					
Female	-	1.55 (0.45–0.99)	-	-	-
Age					
55–64	0.55 (0.25–1.20)	-	-	-	-
65–74	0.54 (0.25–1.18)	1.59 (0.68–3.64)	-	-	-
>75	-	-	-	-	-
Ethnicity					
Black	9.27 (1.50–180)*	-	-	-	-
Chinese	-	-	-	-	-
Asian	3.06 (0.58–23.7)	-	-	-	-
Other	-	-	-	-	-
Education					
GED	-	-	-	9.36 (1.13–65.3) *	-
Certificate	-	-	-	-	-
Associate’s	-	0.40 (0.06–1.50)	-	-	-
Bachelor’s	0.42 (0.16–1.04)	-	-	-	-
Master’s or professional	0.20 (0.05–0.63) **	0.34 (0.02–1.80)	-	-	5.59 (1.71–21.0) **
BMI					
Underweight	-	-	-	-	-
Overweight	-	-	-	-	-
Obese	-	-	-	-	-

* *p*-value <0.05; ** *p*-value <0.01; *** *p*-value <0.001; - Not applicable.

## Data Availability

The data presented in this study are available on request from the corresponding author.
